# Reconstructing Speech from Human Auditory Cortex

**DOI:** 10.1371/journal.pbio.1001251

**Published:** 2012-01-31

**Authors:** Brian N. Pasley, Stephen V. David, Nima Mesgarani, Adeen Flinker, Shihab A. Shamma, Nathan E. Crone, Robert T. Knight, Edward F. Chang

**Affiliations:** 1 Helen Wills Neuroscience Institute, University of California Berkeley, Berkeley, California, United States of America; 2 Institute for Systems Research and Department of Electrical and Computer Engineering, University of Maryland, College Park, Maryland, United States of America; 3 Department of Neurological Surgery, University of California–San Francisco, San Francisco, California, United States of America; 4 Department of Neurology, The Johns Hopkins University, Baltimore, Maryland, United States of America; 5 Department of Psychology, University of California Berkeley, Berkeley, California, United States of America; McGill University, Canada

## Abstract

How the human auditory system extracts perceptually relevant acoustic features of speech is unknown. To address this question, we used intracranial recordings from nonprimary auditory cortex in the human superior temporal gyrus to determine what acoustic information in speech sounds can be reconstructed from population neural activity. We found that slow and intermediate temporal fluctuations, such as those corresponding to syllable rate, were accurately reconstructed using a linear model based on the auditory spectrogram. However, reconstruction of fast temporal fluctuations, such as syllable onsets and offsets, required a nonlinear sound representation based on temporal modulation energy. Reconstruction accuracy was highest within the range of spectro-temporal fluctuations that have been found to be critical for speech intelligibility. The decoded speech representations allowed readout and identification of individual words directly from brain activity during single trial sound presentations. These findings reveal neural encoding mechanisms of speech acoustic parameters in higher order human auditory cortex.

## Abstract 2

Direct brain recordings from neurosurgical patients listening to speech reveal that the acoustic speech signals can be reconstructed from neural activity in auditory cortex.

## Author Summary

Spoken language is a uniquely human trait. The human brain has evolved computational mechanisms that decode highly variable acoustic inputs into meaningful elements of language such as phonemes and words. Unraveling these decoding mechanisms in humans has proven difficult, because invasive recording of cortical activity is usually not possible. In this study, we take advantage of rare neurosurgical procedures for the treatment of epilepsy, in which neural activity is measured directly from the cortical surface and therefore provides a unique opportunity for characterizing how the human brain performs speech recognition. Using these recordings, we asked what aspects of speech sounds could be reconstructed, or decoded, from higher order brain areas in the human auditory system. We found that continuous auditory representations, for example the speech spectrogram, could be accurately reconstructed from measured neural signals. Reconstruction quality was highest for sound features most critical to speech intelligibility and allowed decoding of individual spoken words. The results provide insights into higher order neural speech processing and suggest it may be possible to readout intended speech directly from brain activity.

## Introduction

The early auditory system decomposes speech and other complex sounds into elementary time-frequency representations prior to higher level phonetic and lexical processing [1]–[5]. This early auditory analysis, proceeding from the cochlea to the primary auditory cortex (A1) [1]–[3],[6], yields a faithful representation of the spectro-temporal properties of the sound waveform, including those acoustic cues relevant for speech perception, such as formants, formant transitions, and syllable rate [7]. However, relatively little is known about what specific features of natural speech are represented in intermediate and higher order human auditory cortex. In particular, the posterior superior temporal gyrus (pSTG), part of classical Wernicke's area [8], is thought to play a critical role in the transformation of acoustic information into phonetic and pre-lexical representations [4],[5],[9],[10]. PSTG is believed to participate in an “intermediate” stage of processing that extracts spectro-temporal features essential for auditory object recognition and discards nonessential acoustic features [4],[5],[9]–[11]. To investigate the nature of this auditory representation, we directly quantified how well different stimulus representations account for observed neural responses in nonprimary human auditory cortex, including areas along the lateral surface of STG. One approach, referred to as stimulus reconstruction [12]–[15], is to measure population neural responses to various stimuli and then evaluate how accurately the original stimulus can be reconstructed from the measured responses. Comparison of the original and reconstructed stimulus representation provides a quantitative description of the specific features that can be encoded by the neural population. Furthermore, different stimulus representations, referred to as encoding models, can be directly compared to test hypotheses about how the neural population represents auditory function [16].

In this study, we focus on whether important spectro-temporal auditory features of spoken words and continuous sentences can be reconstructed from population neural responses. Because significant information may be transformed or lost in the course of higher order auditory processing, an exact reconstruction of the physical stimulus is not expected. However, analysis of stimulus reconstruction can reveal the key auditory features that are preserved in the temporal cortex representation of speech. To investigate this, we analyzed multichannel electrode recordings obtained from the surface of human auditory cortex and examined the extent to which these population neural signals could be used for reconstruction of different auditory representations of speech sounds.

## Results

**Figure 1:**
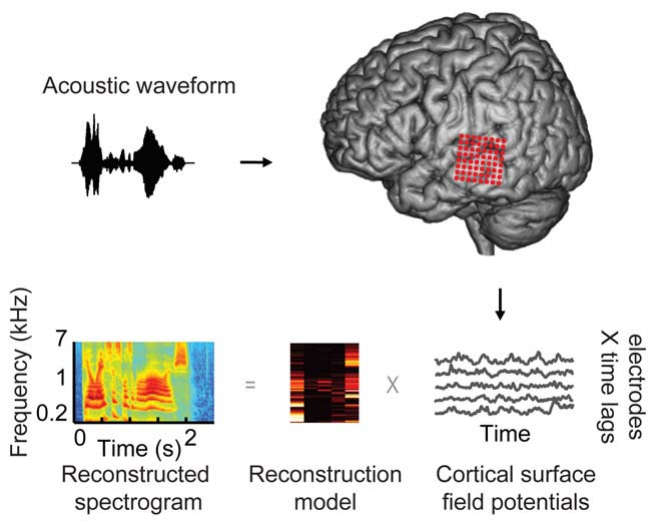
Experiment paradigm. Participants listened to words (acoustic waveform, top left), while neural signals were recorded from cortical surface electrode arrays (top right, red circles) implanted over superior and middle temporal gyrus (STG, MTG). Speech-induced cortical field potentials (bottom right, gray curves) recorded at multiple electrode sites were used to fit multi-input, multi-output models for offline decoding. The models take as input time-varying neural signals at multiple electrodes and output a spectrogram consisting of time-varying spectral power across a range of acoustic frequencies (180–7,000 Hz, bottom left). To assess decoding accuracy, the reconstructed spectrogram is compared to the spectrogram of the original acoustic waveform.

**Figure 2:**
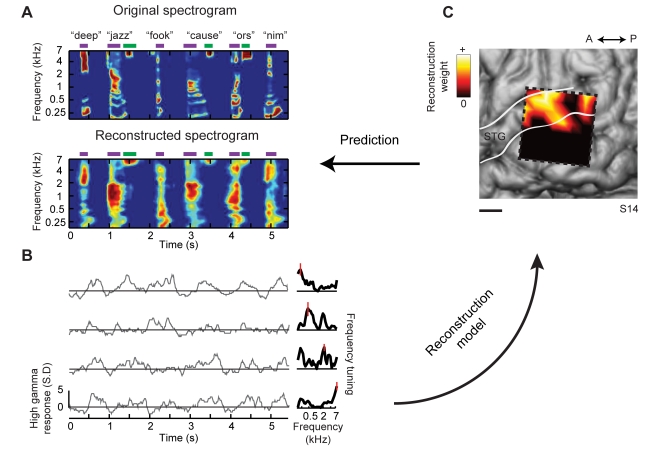
Spectrogram reconstruction. (A) Top: spectrogram of six isolated words (deep, jazz, cause) and pseudowords (fook, ors, nim) presented aurally to an individual participant. Bottom: spectrogram-based reconstruction of the same speech segment, linearly decoded from a set of electrodes. Purple and green bars denote vowels and fricative consonants, respectively, and the spectrogram is normalized within each frequency channel for display. (B) Single trial high gamma band power (70–150 Hz, gray curves) induced by the speech segment in (A). Recordings are from four different STG sites used in the reconstruction. The high gamma response at each site is *z*-scored and plotted in standard deviation (SD) units. Right panel: frequency tuning curves (dark black) for each of the four electrode sites, sorted by peak frequency and normalized by maximum amplitude. Red bars overlay each peak frequency and indicate SEM of the parameter estimate. Frequency tuning was computed from spectro-temporal receptive fields (STRFs) measured at each individual electrode site. Tuning curves exhibit a range of functional forms including multiple frequency peaks (Figures S1B and S2B). (C) The anatomical distribution of fitted weights in the reconstruction model. Dashed box denotes the extent of the electrode grid (shown in Figure 1). Weight magnitudes are averaged over all time lags and spectrogram frequencies and spatially smoothed for display. Nonzero weights are largely focal to STG electrode sites. Scale bar is 10 mm.

Words and sentences from different English speakers were presented aurally to 15 patients undergoing neurosurgical procedures for epilepsy or brain tumor. All patients in this study had normal language capacity as determined by neurological exam. Cortical surface field potentials were recorded from non-penetrating multi-electrode arrays placed over the lateral temporal cortex (Figure 1, red circles), including the pSTG. We investigated the nature of auditory information contained in temporal cortex neural responses using a stimulus reconstruction approach (see Materials and Methods) [12]–[15]. The reconstruction procedure is a multi-input, multi-output predictive model that is fit to stimulus-response data. It constitutes a mapping from neural responses to a multi-dimensional stimulus representation (Figures 1 and 2). This mapping can be estimated using a variety of different learning algorithms [17]. In this study a regularized linear regression algorithm was used to minimize the mean-square error between the original and reconstructed stimulus (see Materials and Methods). Once the model was fit to a training set, it could then be used to predict the spectro-temporal content of any arbitrary sound, including novel speech not used in training.

The key component in the reconstruction algorithm is the choice of stimulus representation, as this choice encapsulates a hypothesis about the neural coding strategy under study. Previous applications of stimulus reconstruction in non-human auditory systems [14],[15] have focused primarily on linear models to reconstruct the auditory spectrogram. The spectrogram is a time-varying representation of the amplitude envelope at each acoustic frequency (Figure 1, bottom left) [18]. The spectrogram envelope of natural sounds is not static but rather fluctuates across both frequency and time [19]–[21]. Envelope fluctuations in the spectrogram are referred to as modulations [18]–[22] and play an important role in the intelligibility of speech [19],[21]. Temporal modulations occur at different temporal rates and spectral modulations occur at different spectral scales. For example, slow and intermediate temporal modulation rates (<4 Hz) are associated with syllable rate, while fast modulation rates (>16 Hz) correspond to syllable onsets and offsets. Similarly, broad spectral modulations relate to vowel formants while narrow spectral structure characterizes harmonics. In the linear spectrogram model, modulations are represented implicitly as the fluctuations of the spectrogram envelope. Furthermore, neural responses are assumed to be linearly related to the spectrogram envelope.

For stimulus reconstruction, we first applied the linear spectrogram model to human pSTG responses using a stimulus set of isolated words from an individual speaker. We used a leave-one-out cross-validation fitting procedure in which the reconstruction model was trained on stimulus-response data from isolated words and evaluated by directly comparing the original and reconstructed spectrograms of the out-of-sample word. Reconstruction accuracy is quantified as the correlation coefficient (Pearson's *r*) between the original and reconstructed stimulus. The reconstruction procedure is illustrated in Figure 2 for one participant with a high-density (4 mm) electrode grid placed over posterior temporal cortex. For different words, the linear model yielded accurate spectrogram reconstructions at the level of single trial stimulus presentations (Figure 2A and B; see Figure S7 and Supporting Audio File S1 for example audio reconstructions). The reconstructions captured major spectro-temporal features such as energy concentration at vowel harmonics (Figure 2A, purple bars) and high frequency components during fricative consonants (Figure 2A, [z] and [s], green bars). The anatomical distribution of weights in the fitted reconstruction model revealed that the most informative electrode sites within temporal cortex were largely confined to pSTG (Figure 2C).

**Figure 3:**
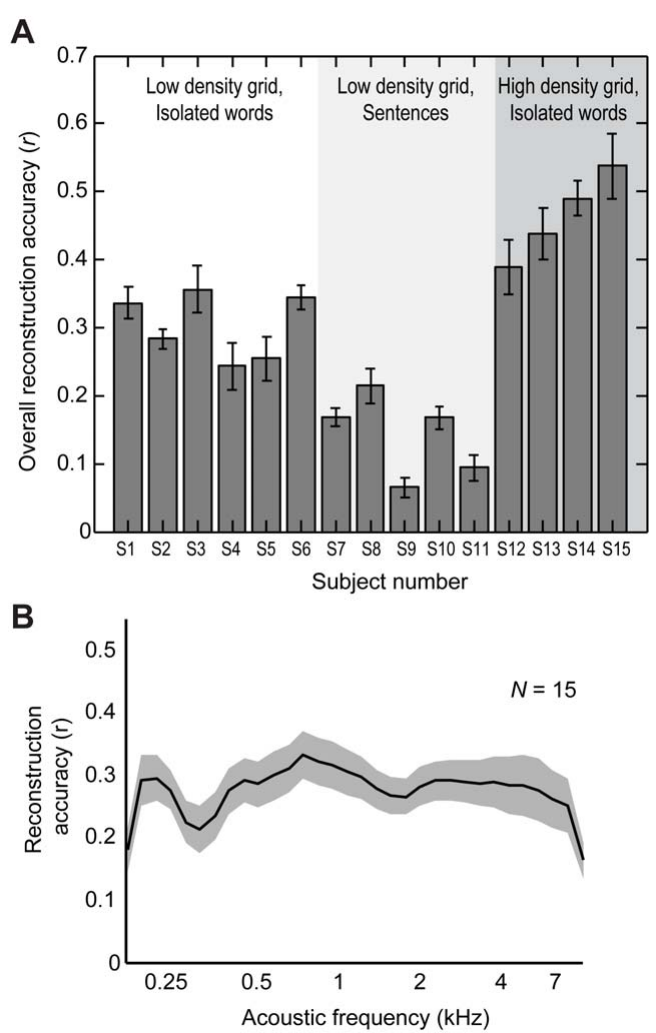
Individual participant and group average reconstruction accuracy. (A) Overall reconstruction accuracy for each participant using the linear spectrogram model. Error bars denote resampling SEM. Overall accuracy is reported as the mean over all acoustic frequencies. Participants are grouped by grid density (low or high) and stimulus set (isolated words or sentences). Statistical significance of the correlation coefficient for each individual participant was computed using a randomization test. Reconstructed trials were randomly shuffled 1,000 times and the correlation coefficient was computed for each shuffle to create a null distribution of coefficients. The *p* value was calculated as the proportion of elements greater than the observed correlation. (B) Reconstruction accuracy as a function of acoustic frequency averaged over all participants (*N* = 15) using the linear spectrogram model. Shaded region denotes SEM over participants.

Across the sample of participants (*N* = 15), cross-validated reconstruction accuracy for single trials was significantly greater than zero in all individual participants (*p*<0.001, randomization test, Figure 3A). At the population level, mean accuracy averaged over all participants and stimulus sets (including different word sets and continuous sentences from different speakers) was highly significant (mean accuracy *r* = 0.28, *p*<10^−5^, one-sample *t* test, *df* = 14). As a function of acoustic frequency, mean accuracy ranged from *r* = ∼0.2–0.3 (Figure 3B).

**Figure 4:**
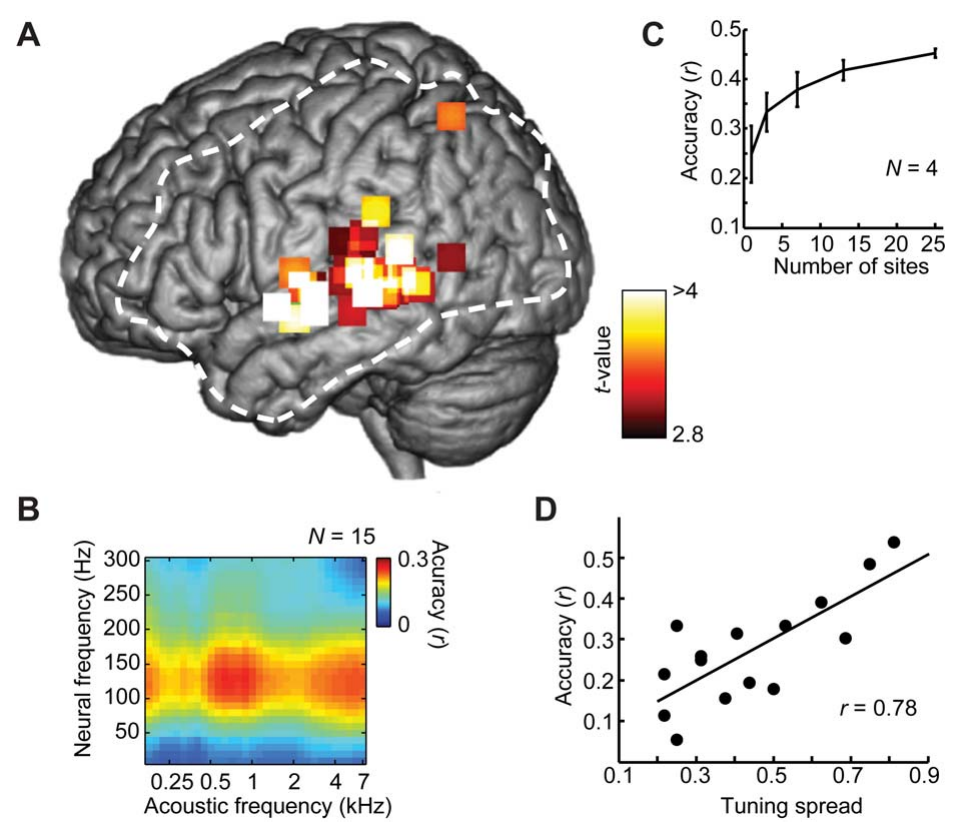
Factors influencing reconstruction quality. (A) Group average *t* value map of informative electrodes, which are predominantly localized to posterior STG. For each participant, informative electrodes are defined as those associated with significant weights (*p*<0.05, FDR correction) in the fitted reconstruction model. To plot electrodes in a common anatomical space, spatial coordinates of significant electrodes are normalized to the MNI (Montreal Neurological Institute) brain template (Yale BioImage Suite, www.bioimagesuite.org). The dashed white line denotes the extent of electrode coverage pooled over participants. (B) Reconstruction accuracy is significantly greater than zero when using neural responses within the high gamma band (∼70–170 Hz; *p*<0.05, one sample *t* tests, *df* = 14, Bonferroni correction). Accuracy was computed separately in 10 Hz bands from 1–300 Hz and averaged across all participants (*N* = 15). (C) Mean reconstruction accuracy improves with increasing number of electrodes used in the reconstruction algorithm. Error bars indicate SEM over 20 cross-validated data sets of four participants with 4 mm high density grids. (D) Accuracy across participants is strongly correlated (*r* = 0.78, *p*<0.001, *df* = 13) with tuning spread (which varied by participant depending on grid placement and electrode density). Tuning spread was quantified as the fraction of frequency bins that included one or more peaks, ranging from 0 (no peaks) to 1 (at least one peak in all frequency bins, ranging from 180–7,000 Hz).

We observed that overall reconstruction quality was influenced by a number of anatomical and functional factors as described below. First, informative temporal electrodes were primarily localized to pSTG. To quantify this, we defined “informative” electrodes as those associated with parameters with high signal-to-noise ratio in the reconstruction models (*t* ratio>2.5, *p*<0.05, false discovery rate (FDR) correction) Figure 4A shows the anatomical distribution of informative electrodes pooled across participants and plotted in standardized anatomical coordinates (Montreal Neurological Institute, MNI) [23]). The distribution was centered in the pSTG (x = −70, y = −29, z = 12, MNI coordinates; Brodmann area 42), and was dispersed along the anterior-posterior axis.

Second, significant predictive power (*r*>0) was largely confined to neural responses in the high gamma band (∼70–170 Hz; Figure 4B; *p*<0.01, one-sample *t* tests, *df* = 14, Bonferroni correction). Predictive power for the high gamma band (∼70–170 Hz) was significantly better compared to other neural frequency bands (*p*<0.05, Bonferroni adjusted pair-wise comparisons between frequency bands, following significant one-way repeated measures analysis of variance (ANOVA), *F*(30,420) = 128.7, *p*<10^−10^). This is consistent with robust speech-induced high gamma responses reported in previous intracranial studies [24]–[29] and with observed correlations between high gamma power and local spike rate [30].

Third, increasing the number of electrodes used in the reconstruction improved overall reconstruction accuracy (Figure 4C). Overall prediction quality was relatively low for participants with five or fewer responsive STG electrodes (mean accuracy *r* = 0.19, *N* = 6 participants) and was robust for cases with high density grids (mean accuracy *r* = 0.43, *N* = 4, mean of 37 responsive STG electrodes per participant).

What neural response properties allow the linear model to find an effective mapping to the stimulus spectrogram? There are two major requirements as described in the following paragraphs. First, individual recording sites must exhibit reliable frequency selectivity (e.g., Figure 2B, right column; Figures S1B, S2). An absence of frequency selectivity (i.e., equal neural response amplitudes to all stimulus frequencies) would imply that neural responses do not encode frequency and could not be used to differentiate stimulus frequencies. To quantify frequency tuning at individual electrodes, we used estimates of standard spectro-temporal receptive fields (STRFs) (see Materials and Methods). The STRF is a forward modeling approach commonly used to estimate neural tuning to a wide variety of stimulus parameters in different sensory systems [16]. We found that different electrodes were sensitive to different acoustic frequencies important for speech sounds, ranging from low (∼200 Hz) to high (∼7,000 Hz). The majority of individual sites exhibited a complex tuning profile with multiple peaks (e.g., Figure 2B, rows 2 and 3; Figure S2B). The full range of the acoustic speech spectrum was encoded by responses from multiple electrodes in the ensemble, although coverage of the spectrum varied by participant (Figure 4D). Across participants, total reconstruction accuracy was positively correlated with the proportion of spectrum coverage (*r* = 0.78, *p*<0.001, *df* = 13; Figure 4D).

**Figure 5:**
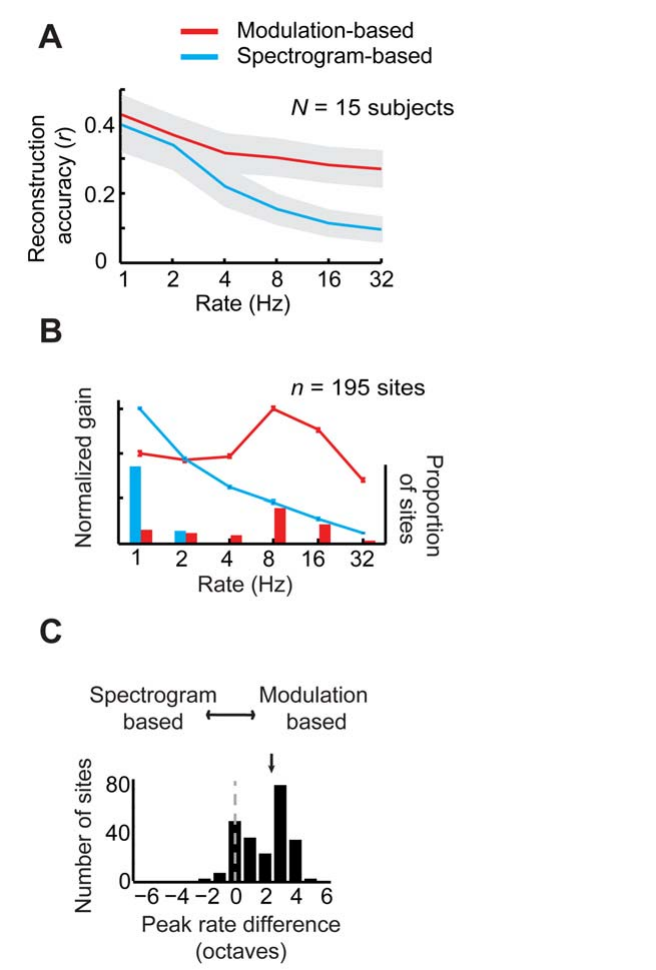
Comparison of linear and nonlinear coding of temporal fluctuations. (A) Mean reconstruction accuracy (*r*) as a function of temporal modulation rate, averaged over all participants (*N* = 15). Modulation-based decoding accuracy (red curve) is higher compared to spectrogram-based decoding (blue curve) for temporal rates ≥4 Hz. In addition, spectrogram-based decoding accuracy is significantly greater than zero for lower modulation rates (≤8 Hz), supporting the possibility of a dual modulation and envelope-based coding scheme for slow modulation rates. Shaded gray regions indicate SEM over participants. (B) Mean ensemble rate tuning curve across all predictive electrode sites (*n* = 195). Error bars indicate SEM. Overlaid histograms indicate proportion of sites with peak tuning at each rate. (C) Within-site differences between modulation and spectrogram-based tuning. Arrow indicates the mean difference across sites. Within-site, nonlinear modulation models are tuned to higher temporal modulation rates than the corresponding linear spectrogram models (*p*<10^−7^, two sample paired *t* test, *df* = 194).

A second key requirement of the linear model is that the neural response must rise and fall reliably with fluctuations in the stimulus spectrogram envelope. This is because the linear model assumes a linear mapping between the response and the spectrogram envelope. This requirement for “envelope-locking” reveals a major limitation of the linear model, which is most evident at fast temporal modulation rates. This limitation is illustrated in Figure 5A (blue curve), which plots reconstruction accuracy as a function of modulation rate. A one-way repeated measures ANOVA (*F*(5,70) = 13.99, *p*<10^−8^) indicated that accuracy was significantly higher for slow modulation rates (≤4 Hz) compared to faster modulation rates (>8 Hz) (*p*<0.05, post hoc pair-wise comparisons, Bonferroni correction). Accuracy for slow and intermediate modulation rates (≤8 Hz) was significantly greater than zero (*r* = ∼0.15 to 0.42; one-sample paired *t* tests, *p*<0.0005, *df* = 14, Bonferroni correction) indicating that the high gamma response faithfully tracks the spectrogram envelope at these rates [26]. However, accuracy levels were not significantly greater than zero at fast modulation rates (>8 Hz; *r* = ∼0.10; one-sample paired *t* tests, *p*>0.05, *df* = 14, Bonferroni correction), indicating a lack of reliable envelope-locking to rapid temporal fluctuations [31].

**Figure 6:**
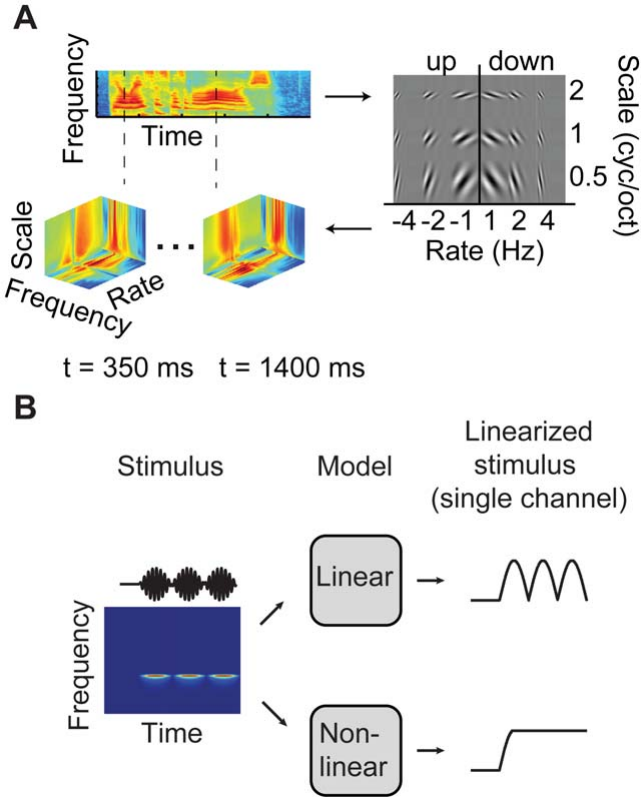
Schematic of nonlinear modulation model. (A) The input spectrogram (top left) is transformed by a linear modulation filter bank (right) followed by a nonlinear magnitude operation (not shown). This nonlinear operation extracts the modulation energy of the incoming spectrogram and generates phase invariance to local fluctuations in the spectrogram envelope. The input representation is the two-dimensional spectrogram S(*f,t*) across frequency *f* and time *t*. The output (bottom left) is the four-dimensional modulation energy representation M(*s,r,f,t*) across spectral modulation scale *s*, temporal modulation rate *r*, frequency *f*, and time *t*. In the full modulation representation [18], negative rates by convention correspond to upward frequency sweeps, while positive rates correspond to downward frequency sweeps. Accuracy for positive and negative rates was averaged unless otherwise shown. See Materials and Methods. (B) Schematic of linear (spectrogram envelope) and nonlinear (modulation energy) temporal coding. Left: acoustic waveform (black curve) and spectrogram of a temporally modulated tone. The linear spectrogram model (top) assumes that neural responses are a linear function of the spectrogram envelope (plotted for the tone center frequency channel, top right). In this case, the instantaneous output may be high or low and does not directly indicate the modulation rate of the envelope. The nonlinear modulation model (bottom) assumes that neural responses are a linear function of modulation energy. This is an amplitude-based coding scheme (plotted for the peak modulation channel, bottom right). The nonlinear modulation model explicitly estimates the modulation rate by taking on a constant value for a constant rate [32].

Given the failure of the linear spectrogram model to reconstruct fast modulation rates, we evaluated competing models of auditory neural encoding. We investigated an alternative, nonlinear model based on modulation (described in detail in [18]). Speech sounds are characterized by both slow and fast temporal modulations (e.g., syllable rate versus onsets) as well as narrow and broad spectral modulations (e.g., harmonics versus formants) [7]. The modulation model represents these multi-resolution features explicitly through a complex wavelet analysis of the auditory spectrogram. Computationally, the modulation representation is generated by a population of modulation-selective filters that analyze the two-dimensional spectrogram and extract modulation energy (a nonlinear operation) at different temporal rates and spectral scales (Figure 6A) [18]. Conceptually, this transformation is similar to the modulus of a 2-D Fourier transform of the spectrogram, localized at each acoustic frequency [18]. The modulation model and applications to speech processing are described in detail in [18] and [7].

The nonlinear component of the model is phase invariance to the spectrogram envelope (Figure 6B). A fundamental difference with the linear spectrogram model is that phase invariance permits a nonlinear temporal coding scheme, whereby envelope fluctuations are encoded by amplitude rather than envelope-locking (Figure 6B). Such amplitude-based coding schemes are broadly referred to as “energy models” [32],[33]. The modulation model therefore represents an auditory analog to the classical energy model of complex cells in the visual system [32]–[36], which are invariant to the spatial phase of visual stimuli.

Reconstructing the modulation representation proceeds similarly to the spectrogram, except that individual reconstructed stimulus components now correspond to modulation energy at different rates and scales instead of spectral energy at different acoustic frequencies (see Materials and Methods, Stimulus Reconstruction). We next compared reconstruction accuracy using the nonlinear modulation model to that of the linear spectrogram model (Figure 5A; Figure S3). In the group data, the nonlinear model yielded significantly higher accuracy compared to the linear model (two-way repeated measures ANOVA; main effect of model type, *F*(1,14) = 33.36, *p*<10^−4^). This included significantly better accuracy for fast temporal modulation rates compared to the linear spectrogram model (4–32 Hz; Figure 5A, red versus blue curves; model type by modulation rate interaction effect, *F*(5,70) = 3.33, *p*<0.01; post hoc pair-wise comparisons, *p*<10^−4^, Bonferroni correction).

The improved performance of the modulation model suggested that this representation provided better neural sensitivity to fast modulation rates compared to the linear spectrogram. To further investigate this possibility, we estimated modulation rate tuning curves at individual STG electrode sites (*n* = 195) using linear and nonlinear STRFs, which are based on the spectrogram and modulation representations, respectively (Figure S4). Consistent with prior recordings from lateral temporal human cortex [31], average envelope-locked responses exhibit prominent tuning to low rates (1–8 Hz) with a gradual loss of sensitivity at higher rates (>8 Hz) (Figure 5B and C). In contrast, the average modulation-based tuning curves preserve sensitivity to much higher rates approaching 32 Hz (Figure 5B and C).

**Figure 7:**
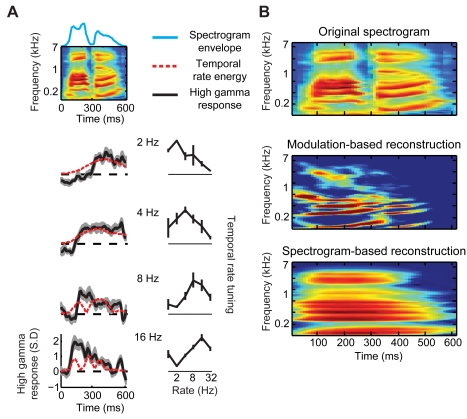
Example of nonlinear modulation coding and reconstruction. (A) Top: the spectrogram of an isolated word (“waldo”) presented aurally to one participant. Blue curve plots the spectrogram envelope, summed over all frequencies. Left panels: induced high gamma responses (black curves, trial averaged) at four different STG sites. Temporal modulation energy of the stimulus (dashed red curves) is overlaid (computed from 2, 4, 8, and 16 Hz modulation filters and normalized to maximum value). Dashed black lines indicate baseline response level. Right panels: nonlinear modulation rate tuning curves for each site (estimated from nonlinear STRFs). Shaded regions and error bars indicate SEM. (B) Original spectrogram (top), modulation-based reconstruction (middle), and spectrogram-based reconstruction (bottom), linearly decoded from a fixed set of STG electrodes. The modulation reconstruction is projected into the spectrogram domain using an iterative projection algorithm and an overcomplete set of modulation filters [18]. The displayed spectrogram is averaged over 100 random initializations of the algorithm.

Sensitivity to fast modulation rates at single STG electrodes is illustrated for one participant in Figure 7A. In this example (the word “waldo”), the spectrogram envelope (blue curve, top) fluctuates rapidly between the two syllables (“wal” and “do,” ∼300 ms). The linear model assumes that neural responses (high gamma power, black curves, left) are envelope-locked and directly track this rapid change. However, robust tracking of such rapid envelope changes was not generally observed, in violation of linear model assumptions. This is illustrated for several individual electrodes in Figure 7A (compare black curves, left, with blue curve, top). In contrast, the modulation representation encodes this fluctuation nonlinearly as an increase in energy at fast rates (>8 Hz, dashed red curves, ∼300 ms, bottom two rows). This allows the model to capture energy-based modulation information in the neural response. Modulation energy encoding at these sites is quantified by the corresponding nonlinear rate tuning curves (Figure 7A, right column). These tuning curves show neural sensitivity to a range of temporal modulations with a single peak rate. For illustrative purposes, Figure 7A (left) compares modulation energy at the peak temporal rate (dashed red curves) with the neural responses (black curves) at each individual site. This illustrates the ability of the modulation model to account for a rapid decrease in the spectrogram envelope without a corresponding decrease in the neural response.

The effect of sensitivity to fast modulation rates can also be observed when the modulation reconstruction is viewed in the spectrogram domain (Figure 7B, middle, see Material and Methods, Reconstruction Accuracy). The result is that dynamic spectral information (such as the upward frequency sweep at ∼400–500 ms, Figure 7B, top) is better resolved compared to the linear spectrogram-based reconstruction (Figure 7B, bottom). These combined results support the idea of an emergent population-level representation of temporal modulation energy in primate auditory cortex [37]. In support of this notion, subpopulations of neurons have been found that exhibit both envelope and energy-based response properties in primary auditory cortex of non-human primates [37]–[39]. This has led to the suggestion of a dual coding scheme in which slow fluctuations are encoded by synchronized (envelope-locked) neurons, while fast fluctuations are encoded by non-synchronized (energy-based) neurons [37].

While these results indicate that a nonlinear model is required to reliably reconstruct fast modulation rates, psychoacoustic studies have shown that slow and intermediate modulation rates (∼1–8 Hz) are most critical for speech intelligibility [19],[21]. These slow temporal fluctuations carry essential phonological information such as formant transitions and syllable rate [7],[19],[21]. The linear spectrogram model, which also yielded good performance within this range (Figure 5A; Figure S3), therefore appears sufficient to reconstruct the essential range of temporal modulations. To examine this issue, we further assessed reconstruction quality by evaluating the ability to identify isolated words using the linear spectrogram reconstructions. We analyzed a participant implanted with a high-density electrode grid (4 mm spacing), the density of which provided a large set of pSTG electrodes. Compared to lower density grid cases, data for this participant included ensemble frequency tuning that covered the majority of the (speech-related) acoustic spectrum (180–7,000 Hz), a factor which we found was critical for accurate reconstruction (Figure 4D). Spectrogram reconstructions were generated for each of 47 words, using neural responses either from single trials or averaged over 3–5 trials per word (same word set and cross-validated fitting procedure as described in Figure 2). To identify individual words from the reconstructions, a simple speech recognition algorithm based on dynamic time warping was used to temporally align words of variable duration [40]. For a target word, a similarity score (correlation coefficient) was then computed between the target reconstruction and the actual spectrograms of each of the 47 words in the candidate set. The 47 similarity scores were sorted and word identification rank was quantified as the percentile rank of the correct word. (1.0 indicates the target reconstruction matched the correct word out of all candidate words; 0.0 indicates the target was least similar to the correct word among all other candidates.) The expected mean of the distribution of identification ranks is 0.5 at chance level.

**Figure 8:**
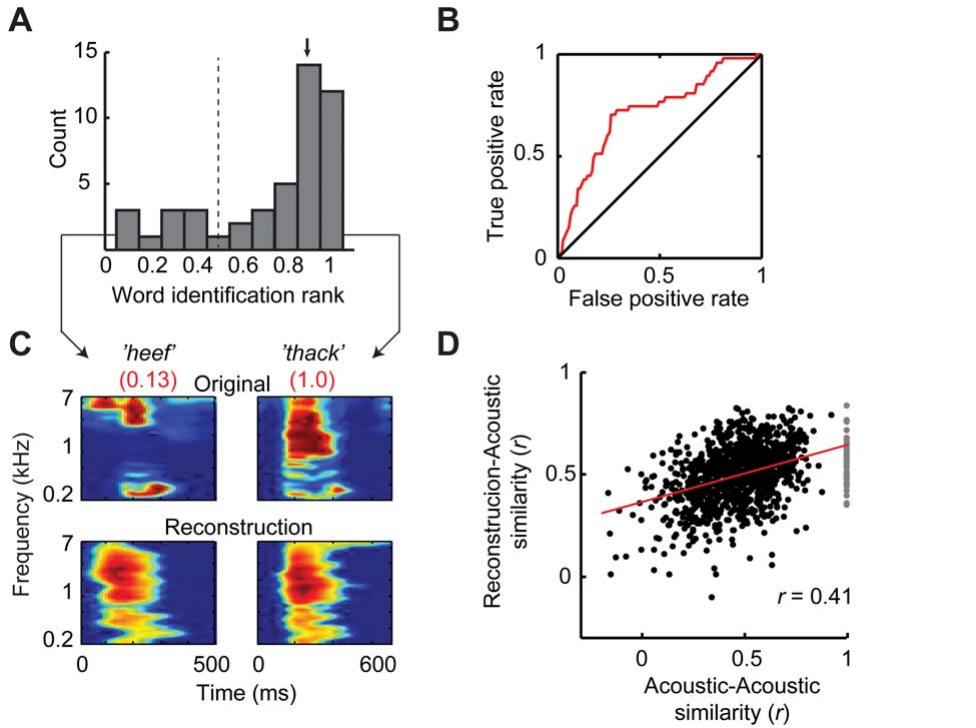
Word identification. Word identification based on the reconstructed spectrograms was assessed using a set of 47 individual words and pseudowords from a single speaker in a high density 4 mm grid experiment. The speech recognition algorithm is described in the text. (A) Distribution of identification rank for all 47 words in the set. Median identification rank is 0.89 (black arrow), which is higher than 0.50 chance level (dashed line; *p*<0.0001; randomization test). Statistical significance was assessed by a randomization test in which a null distribution of the median was constructed by randomly shuffling the word pairs 10,000 times, computing median identification rank for each shuffle, and calculating the percentile rank of the true median in the null distribution. Best performance was achieved after smoothing the spectrograms with a 2-D box filter (500 ms, 2 octaves). (B) Receiver operating characteristic (ROC) plot of identification performance (red curve). Diagonal black line indicates no predictive power. (C) Examples of accurately (right) and inaccurately (left) identified words. Left: reconstruction of pseudoword “heef” is poor and leads to a low identification rank (0.13). Right: reconstruction of pseudoword “thack” is accurate and best matches the correct word out of 46 other candidate words (identification rank = 1.0). (D) Actual and reconstructed word similarity is correlated (*r* = 0.41). Pair-wise similarity between the original spectrograms of individual words is correlated with pair-wise similarity between the reconstructed and original spectrograms. Plotted values are computed prior to spectrogram smoothing used in the identification algorithm. Gray points denote the similarity between identical words.

Word identification using averaged trials was substantially higher than chance (Figure 8A and B, median identification rank = 0.89, *p*<0.0001; randomization test), with correctly identified words exhibiting accurate reconstructions and poorly identified words exhibiting inaccurate reconstructions (Figure 8C). For single trials, identification performance declined slightly but remained significant (median = 0.76, *p*<0.0001; randomization test). In addition, for each possible word pair, we computed the similarity between the two original spectrograms and compared this to the similarity between the reconstructed and actual spectrograms (using averaged trials; Figure 8D; Figure S5). Acoustic and reconstruction word similarities were correlated (*r* = 0.41, *p*<10^−10^, *df* = 45), suggesting that acoustic similarity of the candidate words is likely to influence identification performance (i.e., identification is more difficult when the word set contains many acoustically similar sounds).

## Discussion

These findings demonstrate that key features in continuous and novel speech signals can be accurately reconstructed from STG neural responses using both spectrogram and modulation-based auditory representations, with the latter yielding better predictions at fast temporal modulation rates. For both representations, regions of good prediction performance included the range of spectro-temporal modulations most critical to speech intelligibility [19],[21].

The primary difference between the linear spectrogram and nonlinear modulation models was evident in the predictive accuracy for fast temporal modulations (Figure 5). To understand why the nonlinear modulation model performed better at fast modulation rates, it is useful to consider how the linear and nonlinear models make different assumptions about neural coding. The linear and nonlinear models are specified by different choices of stimulus representation. The linear model assumes a linear mapping between neural responses and the auditory spectrogram. The nonlinear model assumes a linear mapping between neural responses and the modulation representation. The modulation representation itself is a nonlinear transformation of the spectrogram and is based on emergent tuning properties that have been identified in the auditory cortex [18]. Choosing a nonlinear stimulus representation effectively linearizes the stimulus-response mapping and allows one to fit linear models to the new space of transformed stimulus features [17],[35]. If the nonlinear stimulus representation is a more accurate description of neural responses, its predictive accuracy will be higher. In this approach, the choice of stimulus representation for reconstruction encapsulates hypotheses about the coding strategies under study. For example, Rieke et al. [41] reconstructed the sound pressure waveform using neural responses from the bullfrog auditory periphery, where neural responses phase-lock to fluctuations in the raw stimulus waveform [2]. In the central auditory pathway, phase-locking to the stimulus waveform is rare [2], and waveform reconstruction would be expected to fail. Instead, many neurons phase-lock to the spectrogram envelope (a nonlinear transformation of the stimulus waveform) [2]. Consistent with these response properties, spectrogram reconstruction has been demonstrated using neural responses from mammalian primary auditory cortex [14] or the avian midbrain [15]. Beyond primary auditory areas, further processing in intermediate and higher-order auditory cortex likely results in additional stimulus transformations [5]. In this study, we examined human STG, a nonprimary auditory area, and found that a nonlinear modulation representation yielded the best overall reconstruction accuracy, particularly at fast modulation rates (≥4 Hz). This suggests that phase-locking to the amplitude envelope is less robust at higher temporal rates and may instead be coded by an energy-based scheme [37]. Although additional studies are needed, this is consistent with a number of results suggesting that the capacity for envelope-locking decreases along the auditory pathway, extending from the inferior colliculus (32–256 Hz), medial geniculate body (16 Hz), primary auditory cortex (8 Hz), to nonprimary auditory areas (4–8 Hz) [2],[6],[26],[31],[42].

Fidelity of the reconstructions was sufficient to identify individual words using a rudimentary speech recognition algorithm. However, reconstruction quality at present is not clearly intelligible to a human listener (Figure S7 and Supporting Audio File S1). It is possible that a better signal-to-noise ratio or more comprehensive (higher density) recordings in STG could produce intelligible speech reconstructions. Alternatively, the true features represented by STG may not be readily inverted back to an intelligible acoustic waveform. For speech comprehension, it is hypothesized that intermediate and higher-order auditory areas extract or construct information-rich features of speech, while discarding nonessential low-level acoustic information [4],[5],[9],[10],[43]. In the case that STG applies a highly nonlinear stimulus transformation, an exact reconstruction of the acoustic signal from STG responses would not be possible. Instead, speech reconstruction provides an important tool to investigate the critical features that are faithfully represented at different stages of the auditory system. For example, we found that low spectro-temporal modulations (temporal modulations <8 Hz, spectral modulations <4 cycles/octave, Figure S3) are accurately reconstructed from spectrogram or modulation-based models. Modulations within this range correspond to important structural features of natural speech, including formants and syllable rate [19],[21]. Although more work is needed to characterize the neural representation in the STG, this suggests that such key features are preserved at this stage in auditory processing. Our results are therefore consistent with the idea of pSTG as an intermediate stage in a hierarchy of auditory object processing [5],[9],[10],[44].

Hierarchical auditory object processing has been hypothesized to follow a ventral “what” pathway, with an antero-lateral gradient along the superior temporal region [5],[9],[10],[11] where stimulus selectivity increases from pure tones in primary auditory cortex to words and sentences in anterior STG [5]. How can hypotheses about the ventral pathway be tested within the stimulus reconstruction framework? In this framework, encoding models must be developed that encapsulate hypothesized neural mechanisms. These hypotheses are then tested by comparing predictive accuracies of the competing models [16]. For example, in the current work we compared reconstruction accuracy of linear and nonlinear auditory models. An important future direction is to compare performance of these auditory models to higher level models that implement more complex stimulus selectivity. Previous work suggests that categorical representations are an important organizational principle in STG [9],[45]–[47]. These studies found evidence of neural selectivity for entire speech categories, such as vowels or syllables. Unlike the auditory representations studied here, these neural responses were relatively insensitive to acoustic variation. At a more abstract level of representation, a recent functional imaging study also demonstrated that the semantic content of nouns could be used as an effective encoding model across multiple cortical regions [48].

An important application of this approach has also been demonstrated in the study of visual object recognition in the primate visual system [36],[49],[50]. These studies found that structural encoding models, based on spatio-temporal visual features, yielded good performance in primary and intermediate visual areas, including visual areas V1, V2, and V3, whereas a high level encoding model based on semantic features was required to achieve good performance in higher level areas such as V4 and lateral occipital cortex [50]. Our results suggest that a similar approach may be usefully applied to the auditory cortex, where structural auditory models may partially account for responses in primary and intermediate areas (e.g., A1 and pSTG), but development of higher level encoding models could be required to describe more anterior areas in the ventral auditory pathway. As understanding of cortical speech representation improves, future research into speech reconstruction may also be useful for development of neural interfaces for communication, for example by revealing the content of inner speech imagery.

## Materials and Methods

### Participants and Neural Recordings

Electrocorticographic (ECoG) recordings were obtained using subdural electrode arrays implanted in 15 patients undergoing neurosurgical procedures for epilepsy or brain tumor. All participants volunteered and gave their informed consent before testing. The experimental protocol was approved by the Johns Hopkins Hospital, Columbia University Medical Center, University of California, San Francisco and Berkeley Institutional Review Boards and Committees on Human Research. Electrode grids had center-to-center distance of either 4 mm (*N* = 4 participants) [46] or 10 mm (*N* = 11) [24],[25]. Grid placement was determined entirely by clinical criteria and covered left or right fronto-temporal regions in all patients. Localization and coregistration of electrodes with the structural MRI is described in detail in [51]. Multi-channel ECoG data were amplified and digitally recorded with sampling rate = 1,000 Hz (*N* = 6 participants) [24], 2,003 Hz (*N* = 5) [25], or 3,052 Hz (*N* = 4) [46]. All ECoG signals were remontaged to a common average reference [24] after removal of channels with artifacts or excessive noise (including electromagnetic noise from hospital equipment and poor contact with the cortical surface). Time-varying high gamma band power (70–150 Hz) was extracted from the multi-channel ECoG signal using the Hilbert-Huang transform [25], converted to standardized *z*-scores, and used for all analyses (except Figure 4B in which the ECoG signal was filtered into 30 bands of width 10 Hz, ranging from 1–300 Hz, in order to calculate band-specific prediction accuracy). Data from a variety of language tasks were analyzed. Tasks included passive listening (*N* = 5 participants), target word detection (*N* = 5), and word/sentence repetition (*N* = 5).

### Speech Stimuli

Speech stimuli consisted of isolated words from a single speaker (*N* = 10 participants) or sentences from a variety of male and female speakers (*N* = 5). Isolated words included nouns, verbs, proper names, and pseudowords and were recorded by a native English female speaker (0.3–1 s duration, 16 kHz sample rate). Sentences were phonetically transcribed stimuli from the Texas Instruments/Massachusetts Institute of Technology (TIMIT) database (2–4 s, 16 kHz) [52]. Stimuli were presented aurally at the patient's bedside using either external free-field loudspeakers or calibrated ear inserts (Etymotic ER-5A) at approximately 70–80 dB.

The spectrogram representation (linear model) was generated from the speech waveform using a 128 channel auditory filter bank mimicking the auditory periphery [18],[53]. Filters had logarithmically spaced center frequencies ranging from 180–7,000 Hz and bandwidth of approximately 1/12^th^ octave. The spectrogram was subsequently downsampled to 32 frequency channels.

The modulation representation (nonlinear model) was obtained by a 2-D complex wavelet transform of the 128 channel auditory spectrogram [18], implemented by a bank of causal modulation-selective filters spanning a range of spectral scales (0.5–8 cyc/oct) and temporal rates (1–32 Hz). The modulation selective filters are idealized spectro-temporal receptive fields similar to those measured in mammalian primary auditory cortex (Figure 6). The filter bank output constitutes a complex-valued time-varying multi-dimensional speech representation (downsampled to 32 acoustic frequency×12 rate×5 scale = 1,920 total stimulus channels) [18]. The modulation representation is obtained by taking the magnitude of this complex-valued output. In specific analyses (stated in the text), reduced modulation representations were used to reduce dimensionality and to achieve an acceptable computational load, as well as to verify that tuning estimates were not affected by regularization, given the large number of fitted parameters in the full model. Reduced modulation representations included (1) rate-scale (60 total channels) and (2) rate only (six total channels). The rate-scale representation was obtained by averaging along the irrelevant dimension (frequency) prior to the nonlinear magnitude operation. The rate only representation was obtained by filtering the spectrogram with pure temporal modulation filters (described in detail in Chi et al. [18]). Note that spectro-temporal filtering of the spectrogram is directional and captures upward and downward frequency sweeps, which by convention are denoted as positive and negative rates, respectively. Pure temporal filtering in the rate-only representation is not directional and results in half the total number of rate channels. These operations are described in detail in Chi et al. [18]. Figure S6 summarizes the stimulus correlations present in the linear and nonlinear representations.

### Stimulus Reconstruction

The stimulus reconstruction model is the linear mapping between the responses at a set of electrodes and the original stimulus representation (e.g., modulation or spectrogram representation) [12],[14]. For a set of *N* electrodes, we represent the response of electrode *n* at time *t* = 1 … *T* as *R*(*t*, *n*). The reconstruction model, *g*(*τ*, *f*, *n*), is a function that maps *R*(*t*, *n*) to stimulus *S*(*t*, *f*) as follows:
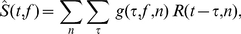
(1)where 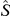 denotes the estimated stimulus representation. Equation 1 implies that the reconstruction of each channel in the stimulus representation, *S_f_*(*t*), from the neural population is independent of the other channels (estimated using a separate set of *g_f_*(*t*, *n*)). If we consider the reconstruction of one such channel, it can be written as:
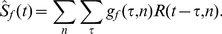
(2)The entire reconstruction function is then described as the collection of functions for each stimulus feature:
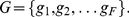
(3)For the spectrogram, time-varying spectral energy in 32 individual frequency channels was reconstructed. For the modulation representation, unless otherwise stated we reconstructed the reduced rate-scale representation, which consists of time-varying modulation energy in 60 rate-scale channels (defined in Speech Stimuli). We used τ = 100 temporal lags, discretized at 10 ms.

### Model Fitting

Prior to model fitting, stimuli and neural response data were synchronized, downsampled to 100 Hz, and standardized to zero mean and unit standard deviation. Model parameters (*G* in Eqn. 3) were fit to a training set of stimulus-response data (ranging from 2.5–17.5 min for different participants) using coordinate gradient descent with early stopping regularization, an iterative linear regression algorithm [16],[36],[49]. Each data set was divided into training (80%), validation (10%), and test sets (10%). Overfitting was minimized by monitoring prediction accuracy on the validation set and terminating the algorithm after a series of 50 iterations failed to improve performance (an indication that overfitting was beginning to occur). Reconstruction accuracy was then evaluated on the independent test set. Coordinate descent produces a sparse solution in the weight vector (i.e., most weight values set to zero) and essentially performs variable selection simultaneously with model fitting [17]. Consequently, there is no requirement to preselect electrodes for the reconstruction model. For grid sizes studied here, inclusion of all electrodes in the reconstruction model can be advantageous because the algorithm encourages irrelevant parameters to maintain zero weight, while allowing the model to capture additional variance using electrodes potentially excluded by feature selection approaches. Equal numbers of parameters are used to estimate each stimulus channel in both linear and nonlinear models. For each stimulus channel, the number of parameters in the corresponding reconstruction filter is *N* electrodes×100 time lags (the number of electrodes for each participant was determined by clinical criteria and therefore *N* varied by participant).

### Cross-Validation

Parameter estimation was performed by a cross-validation procedure using repeated random subsampling [54], also referred to as Monte Carlo cross-validation [55]. This has the advantage over k-fold cross-validation in that the proportion of train/test data is independent of the number of folds. Repeated random sub-sampling is similar to a bootstrap procedure (without replacement) that ensures there is no overlap between training and test data sets. For each repeat, trials were randomly partitioned into training (80% of trials), validation (10%), and test sets (10%); model fitting was then performed using the training/validation data; and reconstruction accuracy was evaluated on the test set. This procedure is repeated multiple times (depending on computational load) and the parameters and reconstruction accuracy measures were averaged over all repeats. The forward encoding models were estimated using 20 resamples; the spectrogram and modulation reconstruction models were estimated using 10 and 3 resamples, respectively (due to increasing computational load). Identical data partitions were used for comparing predictive power for different reconstruction models (i.e., spectrogram versus modulation) to ensure potential differences were not due to different stimuli or noise levels in the evaluation data. To check stability of the generalization error estimates, we verified that estimated spectrogram reconstruction accuracy was stable as a function of the number of resamples used in the estimation (ranging from 3 to 10). The total duration of the test set equaled the length of the concatenated resampled data sets (range of ∼0.8–17.5 min across participants). Standard error of individual parameters was calculated as the standard deviation of the resampled estimates [17]. Statistical significance of individual parameters was assessed by the *t*-ratio (coefficient divided by its resampled standard error estimate). Model fitting was performed with the MATLAB toolbox STRFLab (http://strflab.berkeley.edu).

### Reconstruction Accuracy

Reconstruction accuracy was quantified separately for each stimulus component by computing the correlation coefficient (Pearson's *r*) between the reconstructed and original stimulus component. For each participant, this yielded 32 individual correlation coefficients for the 32 channel spectrogram model and 60 correlation coefficients for the 60 channel rate-scale modulation model (defined in Speech Stimuli). Overall reconstruction accuracy is reported as the mean correlation over all stimulus components.

To make a direct comparison of modulation and spectrogram-based accuracy, the reconstructions need to be compared in the same stimulus space. The linear spectrogram reconstruction was therefore projected into the rate-scale modulation space (using the modulation filterbank as described in Speech Stimuli). This transformation provides an estimate of the modulation content of the spectrogram reconstruction and allows direct comparison with the modulation reconstruction. The transformed reconstruction was then correlated with the 60 rate-scale components of the original stimulus. Accuracy as a function of rate (Figure 5A) was calculated by averaging over the scale dimension. Positive and negative rates were also averaged unless otherwise shown. Comparison of reconstruction accuracy for a subset of data in the full rate-scale-frequency modulation space yielded similar results. To impose additivity and approximate a normal sampling distribution of the correlation coefficient statistic, Fisher's *z*-transform was applied to correlation coefficients prior to tests of statistical significance and prior to averaging over stimulus channels and participants. The inverse z-transform was then applied for all reported mean *r* values.

To visualize the modulation-based reconstruction in the spectrogram domain (Figure 7B), the 4-D modulation representation needs to be inverted [18]. If both magnitude and phase responses are available, the 2-D spectrogram can be restored by a linear inverse filtering operation [18]. Here, only the magnitude response is reconstructed directly from neural activity. In this case, the spectrogram can be recovered approximately from the magnitude-only modulation representation using an iterative projection algorithm and an overcomplete set of modulation filters as described in Chi et al. [18]. Figure 7B displays the average of 100 random initializations of this algorithm. This approach is subject to non-neural errors due to the phase-retrieval problem (i.e., the algorithm does not perfectly recover the spectrogram, even when applied to the original stimulus) [18]. Therefore, quantitative comparisons with the spectrogram-based reconstruction were performed in the modulation space.

Reconstruction accuracy was cross-validated and the reported correlation is the average over all resamples (see Cross-Validation) [53]. Standard error is computed as the standard deviation of the resampled distribution [17]. The reported correlations are not corrected to account for the noise ceiling on prediction accuracy [16], which limits the amount of potentially explainable variance. An ideal model would not achieve perfect prediction accuracy of *r* = 1.0 due to the presence of random noise that is unrelated to the stimulus. With repeated trials of identical stimuli, it is possible to estimate trial-to-trial variability to correct for the amount of potentially explainable variance [56]. In the experiments reported here, a sufficient number of trial repetitions (>5) was generally unavailable for a robust estimate, and uncorrected values are therefore reported.

### STRF Encoding Models

Encoding models describe the linear mapping between the stimulus representation and the neural response at individual sites. For a stimulus representation *s*(*x*,*t*) and instantaneous neural response *r*(*t*) sampled at times *t* = 1 … *T*, the encoding model is defined as the linear mapping [14],[56]:
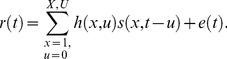
(4)Each coefficient of *h* indicates the gain applied to stimulus feature *x* at time lag *u*. Positive values indicate components of the stimulus correlated with increased neural response, and negative values indicate components correlated with decreased response. The residual, *e*(*t*), represents components of the response (nonlinearities and noise) that cannot be predicted by the encoding model.

Model fitting for the STRF models (*h* in Eqn. 4) proceeded similarly to reconstruction except a standard gradient descent algorithm (with early stopping regularization) was used that does not impose a sparse solution [16],[36],[49]. The linear STRF model included 32 frequency channels×100 time lags (3,200 parameters). The full nonlinear modulation STRF model included 32 frequency×5 scale×12 rate×100 time lags (192,000 parameters) and the reduced rate-time modulation model (Figure S4) included 6 rate×100 time lags (600 parameters). The STRF models were cross-validated using 20 resampled data sets with no overlap between training and test partitions within each resample. Data partitions were identical across STRF model type (linear and nonlinear). We did not enforce identical resampled data sets for estimating STRF and reconstruction models, because the predictive power of these two approaches is not comparable. Tuning curves were estimated from STRFs as follows: Frequency tuning was estimated from the linear STRF models by first setting all inhibitory weights to zero and then summing across the time dimension [53]. Nonlinear rate tuning was estimated from the nonlinear STRF modulation model by the same procedure, using the reduced rate-only representation. Linear rate tuning was estimated from the linear STRF model by filtering the fitted STRF with the modulation filterbank (see Speech Stimuli) and averaging along the irrelevant dimensions. Linear rate tuning computed in this way was similar to that computed from the modulation transfer function (modulus of the 2-D Fourier transform) of the fitted linear STRF [57]. For all tuning curves, standard error was computed as the standard deviation of the resampled estimates [17]. Frequency tuning curve peaks were identified as significant parameters (*t*>2.0) separated by more than a half octave. To calculate ensemble tuning curves (Figure 5B), the tuning curve for each site was normalized by the maximum value and averaged across sites. STG sites with forward prediction accuracy of *r*>0.1 were analyzed (*n* = 195).

## Supporting Information

Figure S1 Anatomical distribution of surface local field potential (LFP) responses and linear STRFs in a low density grid participant (10 mm electrode spacing). (A) Trial averaged spectral LFP responses to English sentences (2–4 s duration) at individual electrode sites. Consistent with previous intracranial language studies [1]–[5], speech stimuli evoke increased high gamma power (∼70–150 Hz) sometimes accompanied by decreased power at lower frequencies (<40 Hz) throughout sites in the temporal auditory cortex. Black outline indicates temporal cortex sites with high gamma responses (>0.5 SD from baseline). (B) Example linear STRFs across all sites for one participant. All models are fit to power in the high gamma band range (70–150 Hz). (C) Anatomical location of subdural electrode grid (10 mm electrode spacing). Yellow outline indicates sites as in (A) and (B).
(TIF)

Figure S2 Frequency tuning. (A) Left panels: linear STRFs for two example electrode sites. Right panels: pure tone frequency tuning (black curves) matches frequency tuning derived from fitted linear STRF models (red curves). For one participant, pure tones (375–6,000 Hz, logarithmically spaced) were presented for 100 ms at 80 dB. Pure tone tuning curves were calculated as the amplitudes of the induced high gamma response across tone frequencies. STRF-derived tuning curves were calculated by first setting all inhibitory weights to zero and then summing across the time dimension [6]. At these two sites, frequency tuning is approximately high-pass (top) or low-pass (bottom). (B) Distribution of the number of frequency tuning peaks across significant electrodes (*N* = 15 participants) estimated from linear STRF models (32-channel). The majority of sites exhibit complex frequency tuning patterns of 2–5 peaks. Peaks were identified as significant parameters (*t*>2.0) separated by more than a half octave.
(TIF)

Figure S3 Mean reconstruction accuracy for the joint rate-scale space across all participants (*N* = 15). Top: modulation-based (nonlinear) decoding accuracy is significantly higher compared to frequency-based (linear) decoding (bottom) for all spectral scales at temporal rates ≥16 Hz (*p*<0.05, post hoc pair-wise comparisons, Bonferroni correction, following significant two-way repeated measures ANOVA; model type by stimulus component interaction effect, *F*(59,826) = 1.84, *p*<0.0005).
(TIF)

Figure S4 Modulation rate tuning was estimated from both linear and nonlinear STRF models, based on the spectrogram or modulation representation, respectively. Linear STRFs have a 2-D parameter space (frequency×time). Modulation rate tuning for the linear STRF was computed by filtering the fitted STRF model with the modulation filterbank (see Materials and Methods) and averaging along the irrelevant dimensions. Modulation rate tuning computed in this way was similar to that computed from the modulation transfer function (MTF) (modulus of the 2-D Fourier transform of the fitted STRF [7]). Nonlinear STRFs have a 4-D parameter space (rate×scale×frequency×time). Modulation-based rate tuning curves were computed by summing across the three irrelevant dimensions [8]. Modulation rate tuning was similar whether this procedure was applied to a reduced dimension model (rate×time only) or to the marginalized full model. Reported estimates of modulation rate tuning were computed from the reduced (rate×time) models. (A) Left: example linear STRF. The linear STRF can be transformed into rate-scale space (the MTF, right) by taking the modulus of the 2-D Fourier transform [7] or by filtering the STRF with the modulation filter bank. The linear modulation rate tuning curve (blue curve, top) is obtained after averaging along the scale dimension. (B) Left: example nonlinear STRF from the same site as in (A), fit in the rate-time parameter space. Right: the corresponding modulation-based rate tuning curve (red) is plotted against the spectrogram-based tuning curve (blue) from (A) (only positive rates are shown).
(TIF)

Figure S5 Confusion matrix for word identification (Figure 8). Left: pair-wise similarities (correlation coefficient) between actual auditory spectrograms of each word pair. Right: pair-wise similarities between reconstructed and actual spectrograms of each word pair. Correlations were computed prior to any spectrogram smoothing.
(TIF)

Figure S6 Stimulus correlations in linear and nonlinear stimulus representations. Speech, like other natural sounds, has strong stimulus correlations (illustrated for acoustic frequency, top panels, and temporal modulation rate, bottom panels). Correlations were estimated from 1,000 randomly selected TIMIT sentences at different time lags (*τ* = 0, 50, 250 ms; note the temporal asymmetry due to the use of causal modulation filters). Under an efficient coding hypothesis [9], these statistical redundancies may be exploited by the brain during sensory processing. In this study, we used an optimal linear estimator (Wiener filter) [10], which is essentially a multivariate linear regression and does not account for correlations among the output variables. Stimulus reconstruction therefore reflects an upper bound on the stimulus features that are encoded by the neural ensemble [10]. As described in previous work [10],[11], the effect of stimulus statistics on reconstruction accuracy can be explored systematically using different stimulus priors.
(TIF)

Figure S7 Audio playback of reconstructed speech. The audio file contains a sequence of six isolated words that were reconstructed from single trial neural activity. Single trial reconstructions are generally not intelligible. However, coarse features such as syllable structure may be discerned. In addition, up and down frequency sweeps (corresponding to faster temporal rates) are more evident in the modulation reconstructions compared to the spectrogram reconstructions. Perceptual similarities between original and reconstructed words can be more easily recognized after first listening to the original sound. In the audio file, each word is presented as a sequence of the original sound heard by the participant, followed by the spectrogram (linear) reconstruction, followed by the modulation (nonlinear) reconstruction. The figure shows the spectrograms of the original and reconstructed words. For audio playback, the spectrogram or modulation representations must be converted to an acoustic waveform, a transformation that requires both magnitude and phase information. Because the reconstructed representations are magnitude-only, the phase must be estimated. In general, this is known as the phase retrieval problem [8]. To recover the acoustic waveform from the spectrogram, we used an iterative projection algorithm to estimate the phase [8]. This step introduces additional acoustic artifacts that can distort the auditory features reconstructed directly from neural responses. Consequently, the audio file is an accurate but not perfect reflection of the reconstructed speech representation. A similar algorithm can be used to recover the spectrogram from the modulation representation [8]. For the purposes of this demo, we instead projected the spectrogram reconstruction into the (complex) modulation domain, extracted the phase, and then combined the extracted phase with the reconstructed magnitude of the modulation representation. With both phase and magnitude information, an invertible transformation can then be used to convert the (complex) modulation representation back to the spectrogram [8]. Finally, to aid perceptual inspection of the reconstructions, the sample rate of the audio file is slightly slower (14 kHz) than that presented to participants (16 kHz).
(TIF)

Text S1 Supporting Information references.
(PDF)

Audio File S1 Example audio of reconstructed speech.
(WAV)

## Abbreviations

A1: primary auditory cortex
STG: superior temporal gyrus
STRF: spectro-temporal receptive field
