# Supplementary figures and images for: Reconstructing Speech from Human Auditory Cortex
Source: PLoS Biol. 2012 Jan 31;10(1):e1001251. doi: 10.1371/journal.pbio.1001251 (PMC3269422; doi:10.1371/journal.pbio.1001251)

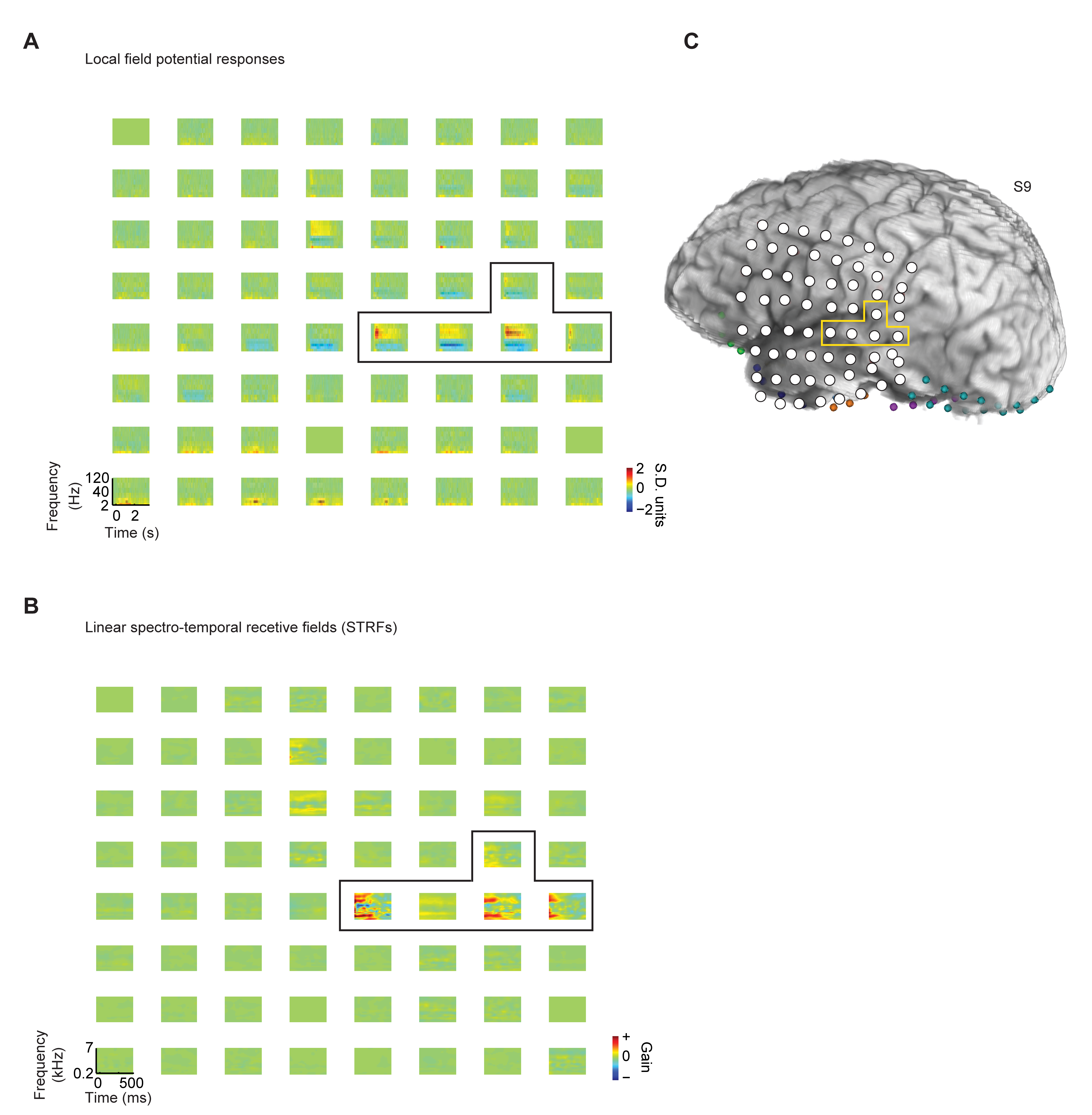

Supplement: Figure S1 — Anatomical distribution of surface local field potential (LFP) responses and linear STRFs in a low density grid participant (10 mm electrode spacing). (A) Trial averaged spectral LFP responses to English sentences (2–4 s duration) at individual electrode sites. Consistent with previous intracranial language studies [1]–[5], speech stimuli evoke increased high gamma power (∼70–150 Hz) sometimes accompanied by decreased power at lower frequencies (<40 Hz) throughout sites in the temporal auditory cortex. Black outline indicates temporal cortex sites with high gamma responses (>0.5 SD from baseline). (B) Example linear STRFs across all sites for one participant. All models are fit to power in the high gamma band range (70–150 Hz). (C) Anatomical location of subdural electrode grid (10 mm electrode spacing). Yellow outline indicates sites as in (A) and (B). (TIF) [file pbio.1001251.s001.tif]

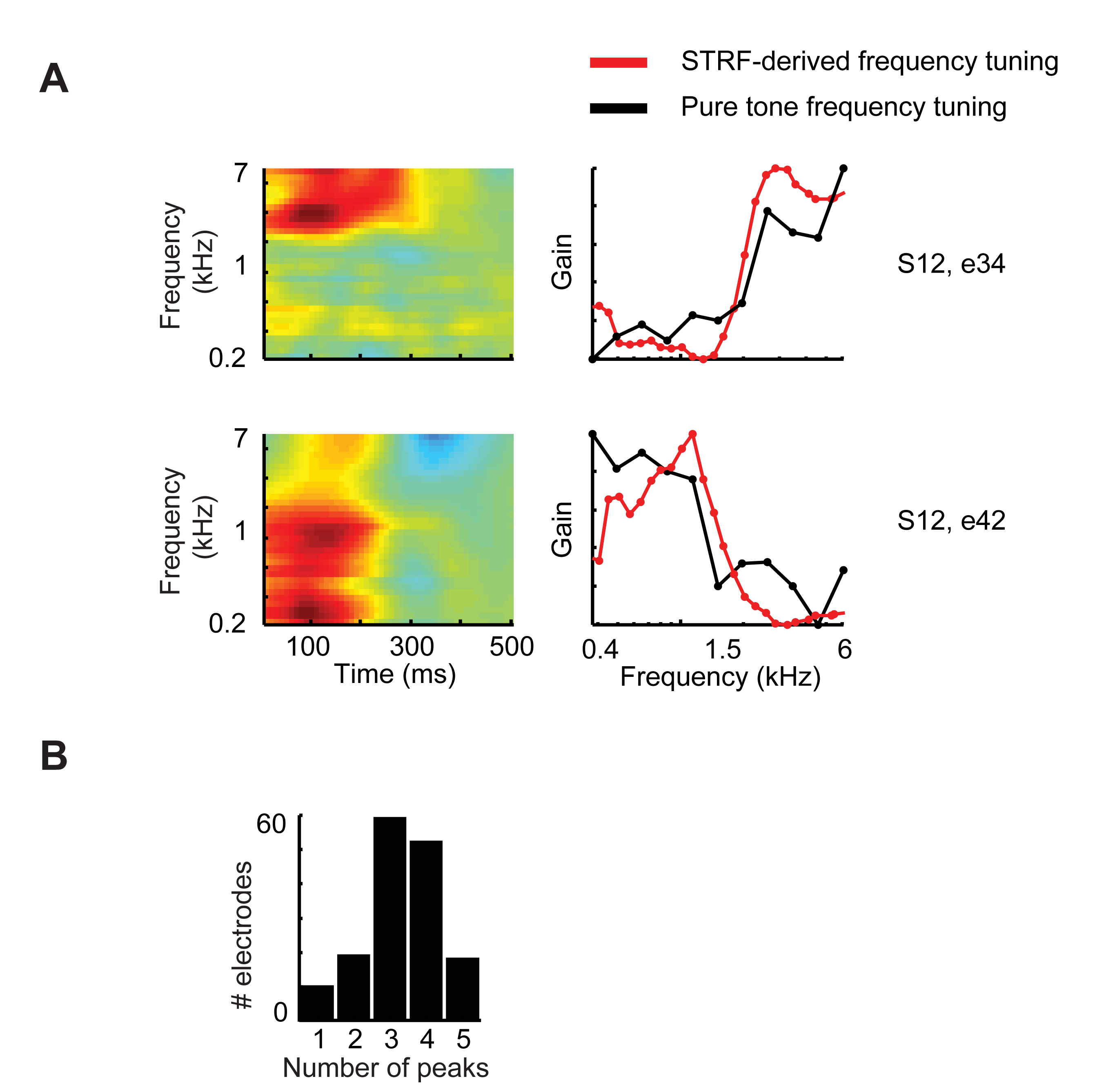

Supplement: Figure S2 — Frequency tuning. (A) Left panels: linear STRFs for two example electrode sites. Right panels: pure tone frequency tuning (black curves) matches frequency tuning derived from fitted linear STRF models (red curves). For one participant, pure tones (375–6,000 Hz, logarithmically spaced) were presented for 100 ms at 80 dB. Pure tone tuning curves were calculated as the amplitudes of the induced high gamma response across tone frequencies. STRF-derived tuning curves were calculated by first setting all inhibitory weights to zero and then summing across the time dimension [6]. At these two sites, frequency tuning is approximately high-pass (top) or low-pass (bottom). (B) Distribution of the number of frequency tuning peaks across significant electrodes (N = 15 participants) estimated from linear STRF models (32-channel). The majority of sites exhibit complex frequency tuning patterns of 2–5 peaks. Peaks were identified as significant parameters (t>2.0) separated by more than a half octave. (TIF) [file pbio.1001251.s002.tif]

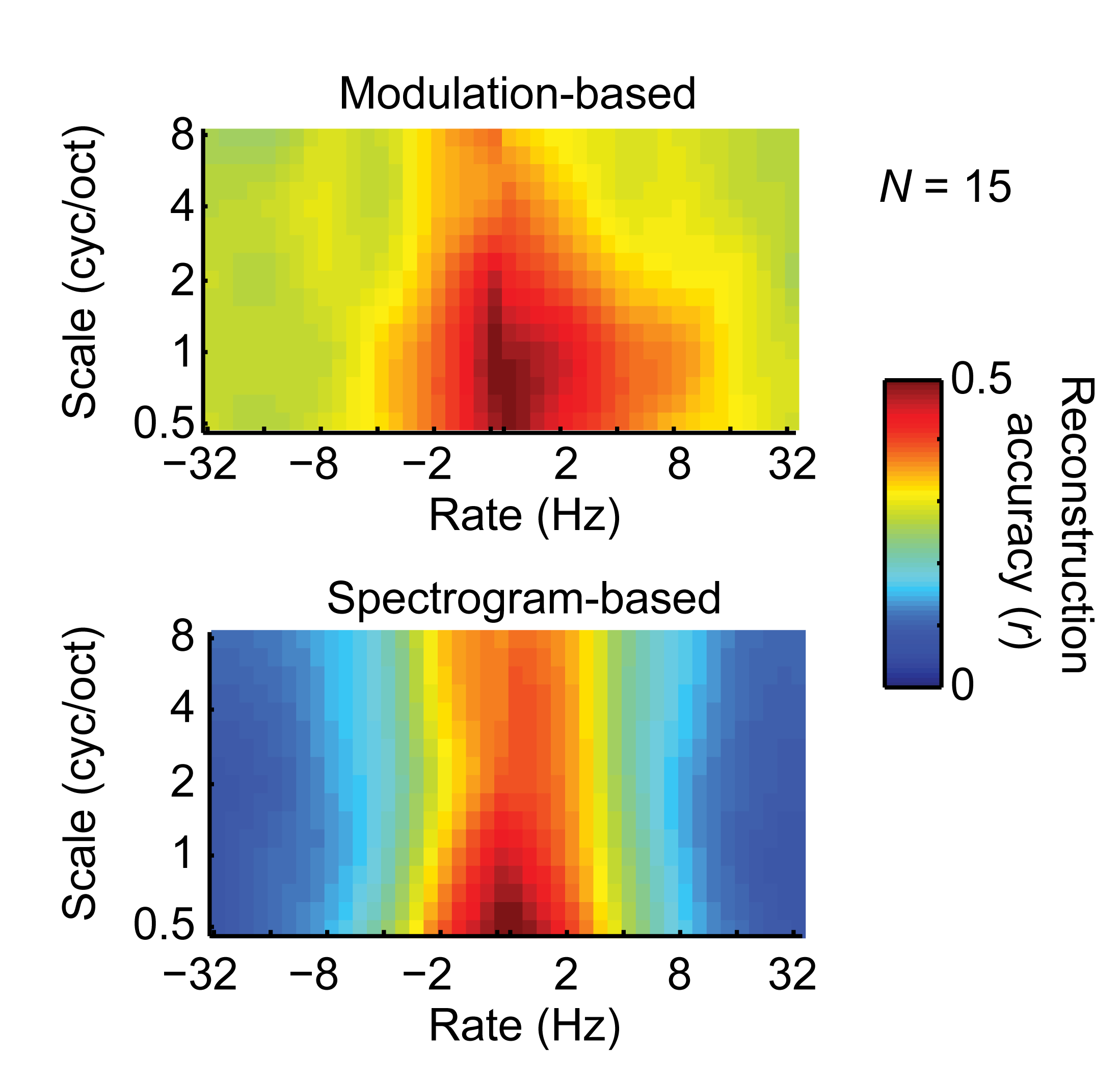

Supplement: Figure S3 — Mean reconstruction accuracy for the joint rate-scale space across all participants (N = 15). Top: modulation-based (nonlinear) decoding accuracy is significantly higher compared to frequency-based (linear) decoding (bottom) for all spectral scales at temporal rates ≥16 Hz (p<0.05, post hoc pair-wise comparisons, Bonferroni correction, following significant two-way repeated measures ANOVA; model type by stimulus component interaction effect, F(59,826) = 1.84, p<0.0005). (TIF) [file pbio.1001251.s003.tif]

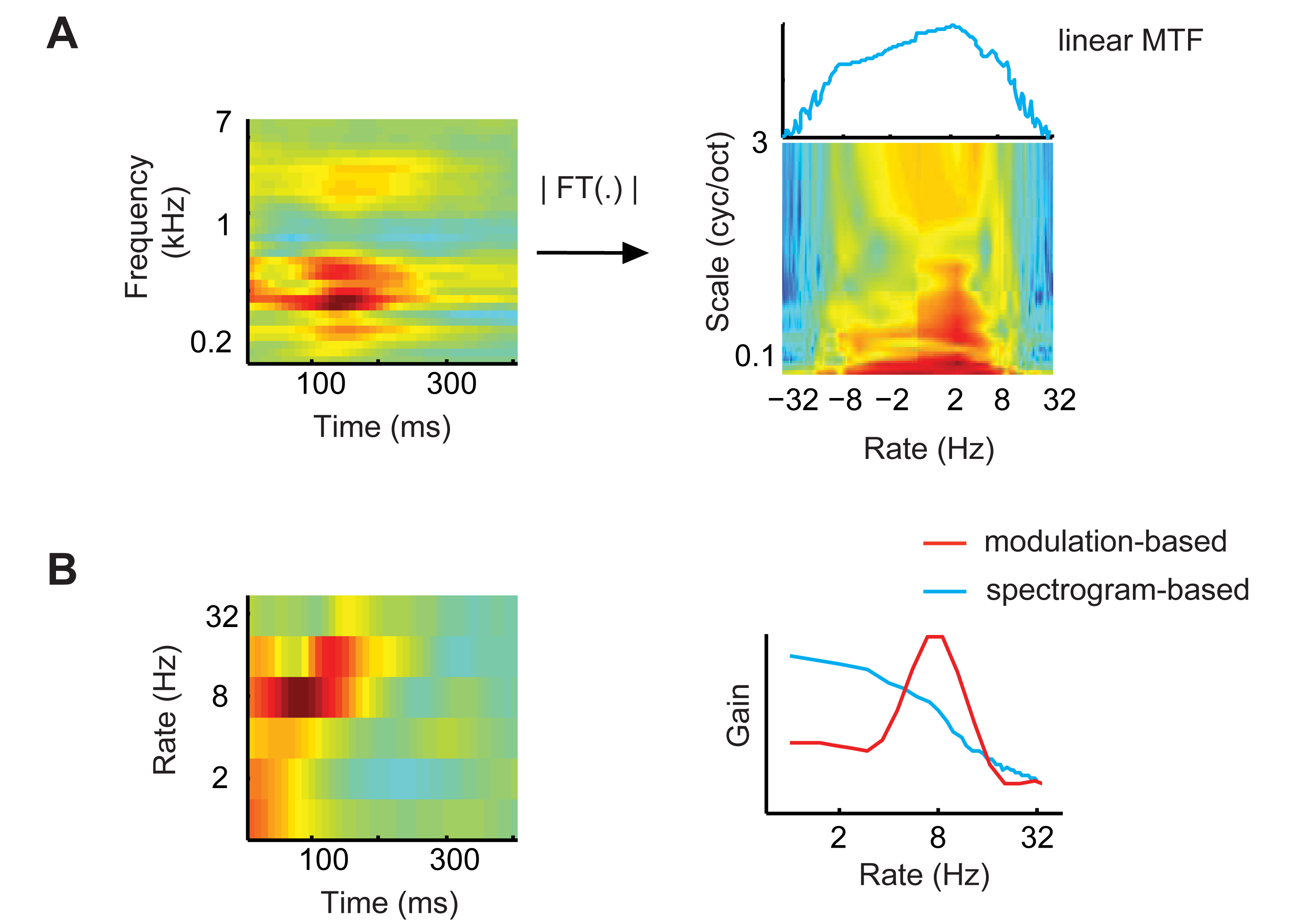

Supplement: Figure S4 — Modulation rate tuning was estimated from both linear and nonlinear STRF models, based on the spectrogram or modulation representation, respectively. Linear STRFs have a 2-D parameter space (frequency×time). Modulation rate tuning for the linear STRF was computed by filtering the fitted STRF model with the modulation filterbank (see Materials and Methods) and averaging along the irrelevant dimensions. Modulation rate tuning computed in this way was similar to that computed from the modulation transfer function (MTF) (modulus of the 2-D Fourier transform of the fitted STRF [7]). Nonlinear STRFs have a 4-D parameter space (rate×scale×frequency×time). Modulation-based rate tuning curves were computed by summing across the three irrelevant dimensions [8]. Modulation rate tuning was similar whether this procedure was applied to a reduced dimension model (rate×time only) or to the marginalized full model. Reported estimates of modulation rate tuning were computed from the reduced (rate×time) models. (A) Left: example linear STRF. The linear STRF can be transformed into rate-scale space (the MTF, right) by taking the modulus of the 2-D Fourier transform [7] or by filtering the STRF with the modulation filter bank. The linear modulation rate tuning curve (blue curve, top) is obtained after averaging along the scale dimension. (B) Left: example nonlinear STRF from the same site as in (A), fit in the rate-time parameter space. Right: the corresponding modulation-based rate tuning curve (red) is plotted against the spectrogram-based tuning curve (blue) from (A) (only positive rates are shown). (TIF) [file pbio.1001251.s004.tif]

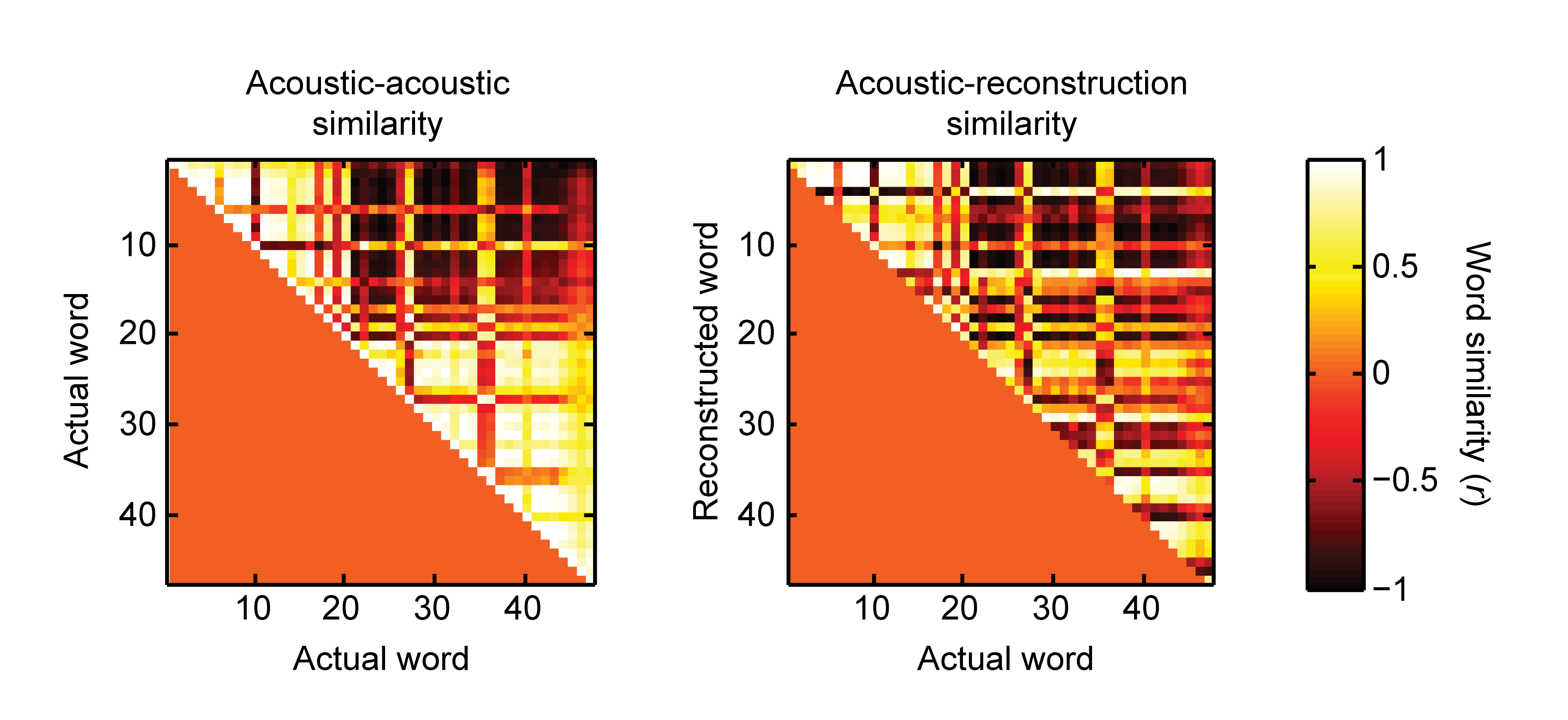

Supplement: Figure S5 — Confusion matrix for word identification (Figure 8). Left: pair-wise similarities (correlation coefficient) between actual auditory spectrograms of each word pair. Right: pair-wise similarities between reconstructed and actual spectrograms of each word pair. Correlations were computed prior to any spectrogram smoothing. (TIF) [file pbio.1001251.s005.tif]

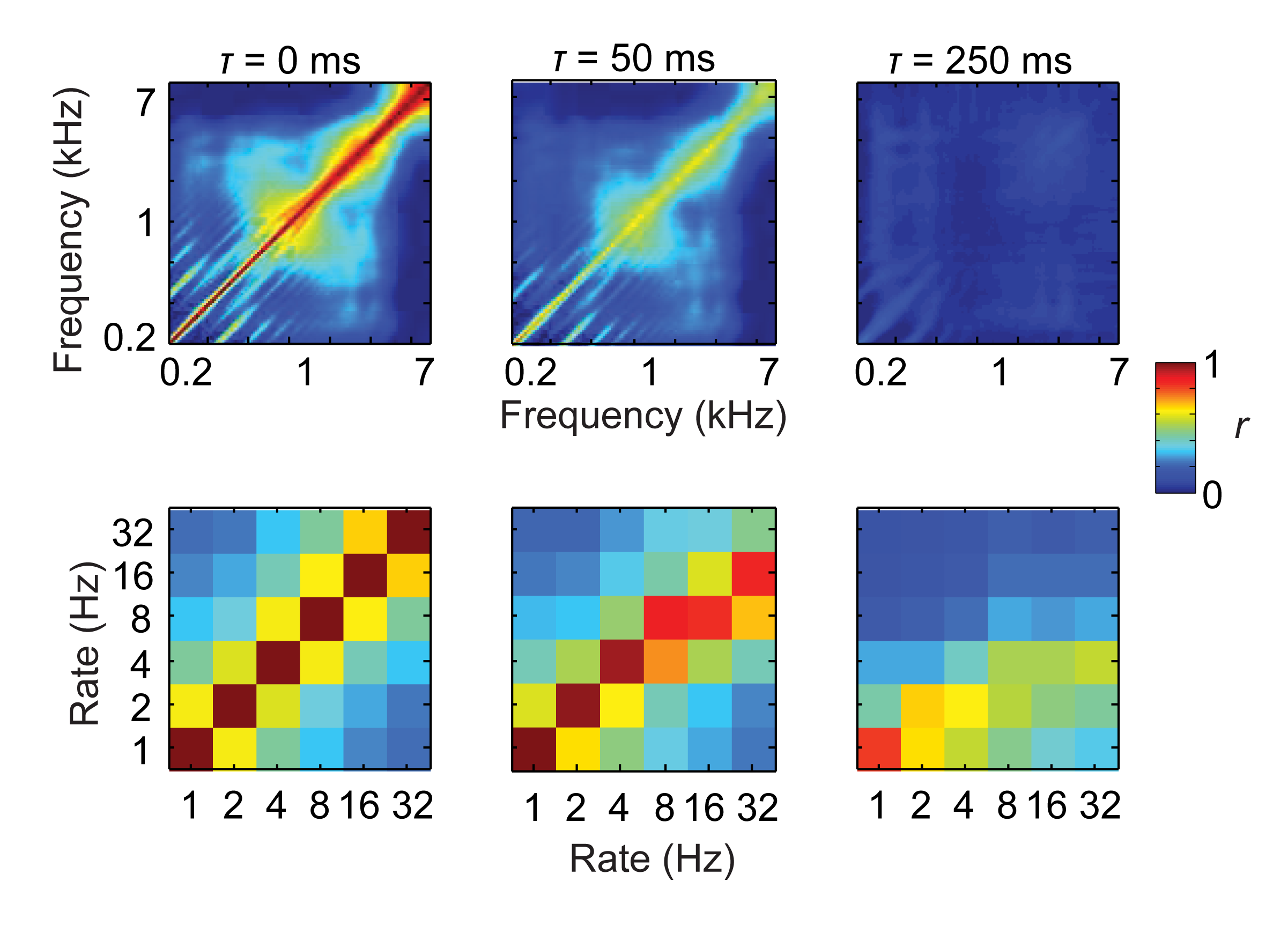

Supplement: Figure S6 — Stimulus correlations in linear and nonlinear stimulus representations. Speech, like other natural sounds, has strong stimulus correlations (illustrated for acoustic frequency, top panels, and temporal modulation rate, bottom panels). Correlations were estimated from 1,000 randomly selected TIMIT sentences at different time lags (τ = 0, 50, 250 ms; note the temporal asymmetry due to the use of causal modulation filters). Under an efficient coding hypothesis [9], these statistical redundancies may be exploited by the brain during sensory processing. In this study, we used an optimal linear estimator (Wiener filter) [10], which is essentially a multivariate linear regression and does not account for correlations among the output variables. Stimulus reconstruction therefore reflects an upper bound on the stimulus features that are encoded by the neural ensemble [10]. As described in previous work [10],[11], the effect of stimulus statistics on reconstruction accuracy can be explored systematically using different stimulus priors. (TIF) [file pbio.1001251.s006.tif]

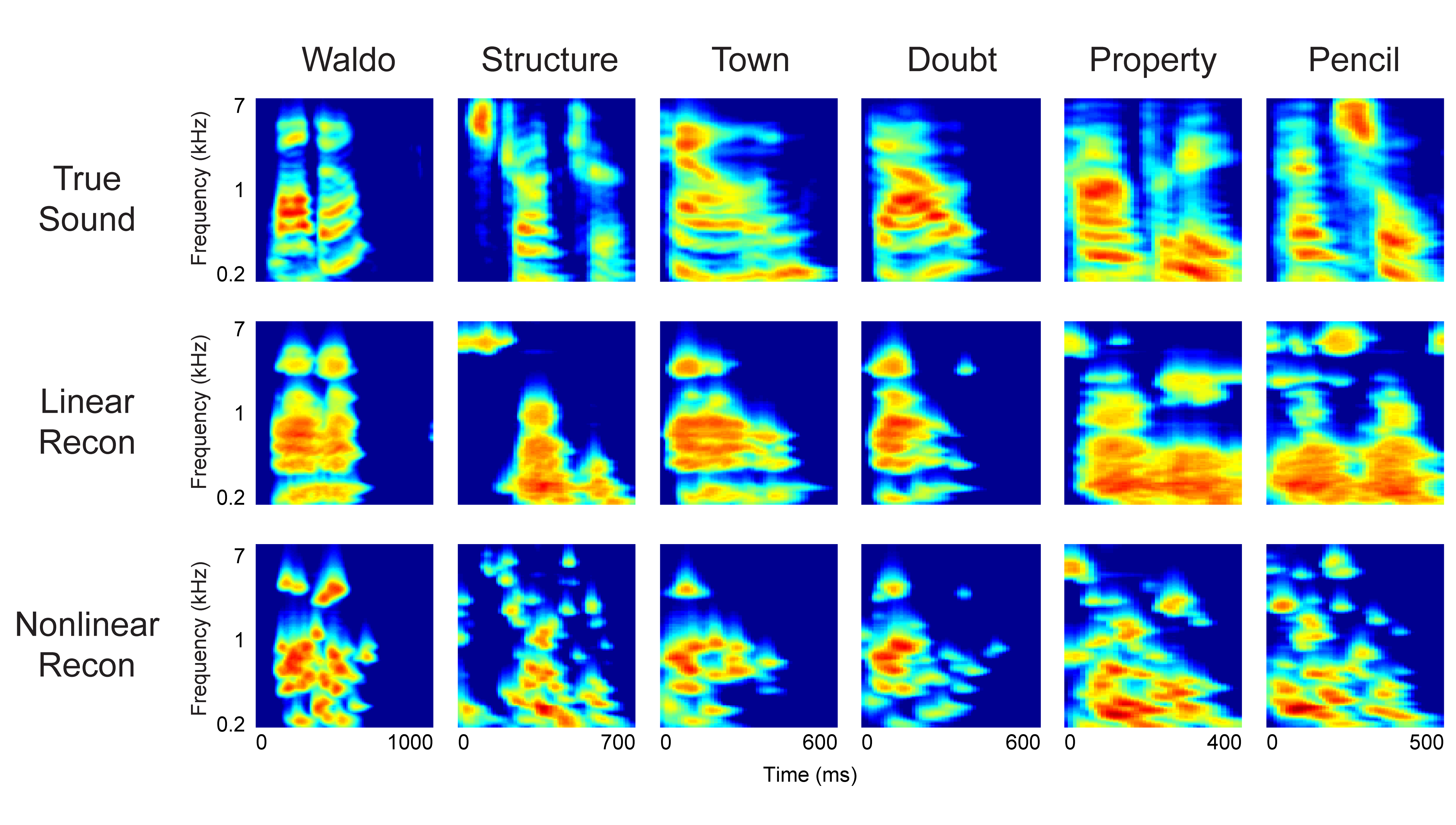

Supplement: Figure S7 — Audio playback of reconstructed speech. The audio file contains a sequence of six isolated words that were reconstructed from single trial neural activity. Single trial reconstructions are generally not intelligible. However, coarse features such as syllable structure may be discerned. In addition, up and down frequency sweeps (corresponding to faster temporal rates) are more evident in the modulation reconstructions compared to the spectrogram reconstructions. Perceptual similarities between original and reconstructed words can be more easily recognized after first listening to the original sound. In the audio file, each word is presented as a sequence of the original sound heard by the participant, followed by the spectrogram (linear) reconstruction, followed by the modulation (nonlinear) reconstruction. The figure shows the spectrograms of the original and reconstructed words. For audio playback, the spectrogram or modulation representations must be converted to an acoustic waveform, a transformation that requires both magnitude and phase information. Because the reconstructed representations are magnitude-only, the phase must be estimated. In general, this is known as the phase retrieval problem [8]. To recover the acoustic waveform from the spectrogram, we used an iterative projection algorithm to estimate the phase [8]. This step introduces additional acoustic artifacts that can distort the auditory features reconstructed directly from neural responses. Consequently, the audio file is an accurate but not perfect reflection of the reconstructed speech representation. A similar algorithm can be used to recover the spectrogram from the modulation representation [8]. For the purposes of this demo, we instead projected the spectrogram reconstruction into the (complex) modulation domain, extracted the phase, and then combined the extracted phase with the reconstructed magnitude of the modulation representation. With both phase and magnitude information, an invertibl [file pbio.1001251.s007.tif]
