# Supplementary material for: Reconstructing Speech from Human Auditory Cortex
Source: PLoS Biol. 2012 Jan 31;10(1):e1001251. doi: 10.1371/journal.pbio.1001251 (PMC3269422; doi:10.1371/journal.pbio.1001251)
Supplement: Text S1 — Supporting Information references. (PDF) [file pbio.1001251.s008.pdf]

Supporting Information References

1. Canolty RT, Soltani M, Dalal SS, Edwards E, Dronkers NF, et al. (2007) Spatiotemporal dynamics of word processing in the human brain. Front Neurosci 1: 185-196.
2. Crone NE, Boatman D, Gordon B, Hao L (2001) Induced electrocorticographic gamma activity during auditory perception. Brazier Award-winning article, 2001. Clin Neurophysiol 112: 565-582.
3. Edwards E, Soltani M, Kim W, Dalal SS, Nagarajan SS, et al. (2009) Comparison of time-frequency responses and the event-related potential to auditory speech stimuli in human cortex. J Neurophysiol 102: 377-386.
4. Nourski KV, Reale RA, Oya H, Kawasaki H, Kovach CK, et al. (2009) Temporal envelope of time-compressed speech represented in the human auditory cortex. J Neurosci 29: 15564-15574.
5. Pei X, Leuthardt EC, Gaona CM, Brunner P, Wolpaw JR, et al. (2011) Spatiotemporal dynamics of electrocorticographic high gamma activity during overt and covert word repetition. Neuroimage 54: 2960-2972.
6. David SV, Mesgarani N, Shamma SA (2007) Estimating sparse spectro-temporal receptive fields with natural stimuli. Network 18: 191-212.
7. Miller LM, Escabi MA, Read HL, Schreiner CE (2002) Spectrotemporal receptive fields in the lemniscal auditory thalamus and cortex. J Neurophysiol 87: 516-527.
8. Chi T, Ru P, Shamma SA (2005) Multiresolution spectrotemporal analysis of complex sounds. J Acoust Soc Am 118: 887-906.
9. Barlow HB (1972) Single units and sensation: a neuron doctrine for perceptual psychology? Perception 1: 371-394.
10. Mesgarani N, David SV, Fritz JB, Shamma SA (2009) Influence of context and behavior on stimulus reconstruction from neural activity in primary auditory cortex. J Neurophysiol 102: 3329-3339.
11. Ramirez AD, Ahmadian Y, Schumacher J, Schneider D, Woolley SM, et al. (2011) Incorporating naturalistic correlation structure improves spectrogram reconstruction from neuronal activity in the songbird auditory midbrain. J Neurosci 31: 3828-3842.
